# Oncocytic carcinoma of the salivary gland with thymoma: A case report and review of the literature

**DOI:** 10.3892/ol.2014.2707

**Published:** 2014-11-19

**Authors:** YONGCHENG CAO, MING ZHU, RUIQI MAO, RUIXUE CAO, GUOLI YU, AIJUN NIU

**Affiliations:** 1 Department of Pathology, General Hospital of Jinan Military Command, Jinan, Shandong 250031, P.R. China; 2 Department of Radiology, General Hospital of Jinan Military Command, Jinan, Shandong 250031, P.R. China; 3 Department of Laboratory Medicine, General Hospital of Jinan Military Command, Jinan, Shandong 250031, P.R. China

**Keywords:** oncocytic carcinoma, salivary, thymoma, PTAH stain

## Abstract

Oncocytic carcinoma (OC) arising in the salivary gland is a very rare tumor with only 32 previously reported cases. In this report, we describe a novel case of oncocytic carcinoma with associated thymoma, which arose in the left parotid gland of a 66-year-old male with a history of a painless left parotid mass for 1 year. Oncocytes are large, polygonal cells that are characterized by marked cellular atypia, frequent mitoses, wide eosinophilic granular cytoplasm, a central nucleus and a prominent nucleolus. The follow-up data showed no evidence of recurrence and the patient is in a good health 20 months after the surgery. In the current case, the patient had not only OC but also thymoma, which is exceedingly rare and may represent the first documented case in the literature.

## Introduction

Oncocytic carcinomas (OCs) are very rare neoplasms that have been reported to occur in the nasal and thoracic cavities, ovary, kidney, thyroid gland, salivary gland, breast and parathyroid (1,2). These tumors represent 11% of all oncocytic salivary gland neoplasms, 0.5% of all epithelial salivary gland malignancies and 0.18% of all epithelial salivary gland tumors (1). Bauer and Bauer (3) reported the first case in 1953, and, in total, only 31 cases have been reported in English-language literature (4–22).

Oncocytes are typically large epithelial cells with a low nuclear-to-cytoplasmic ratio, a centrally situated round nucleus with a prominent nucleolus and an abundant bright eosinophilic granular cytoplasm that is ultrastructurally characterized by numerous mitochondria (14). Thymic epithelial tumors include thymomas and thymic carcinomas. Although occurence is rare (accounting for 0.2–1.5% of all malignancies) (23), they present the most common tumor of the anterior mediastinum (24). Thymomas are neoplasms arising from or exhibiting differentiation towards thymic epithelial cells. Thymomas are classified into two major types depending on whether the neoplastic epithelial cells have an oval shape and are uniformly bland (type A thymoma) or whether the cells have a predominantly round or polygonal appearance (type B thymoma). Thymomas which exhibit type A and B-like features are classified as type AB (25). In this case report, the patient not only had OC but also thymoma. To the best of our knowledge, this is the first reported case of an OC patient exhibiting type AB thyoma. Written informed consent was obtained from the patient.

## Case report

### Case presentation

A 66-year-old male was admitted to the General Hospital of Jinan Military Command (Jinan, China) with a 1-year history of a painless left parotid mass that was gradually increasing in size. Physical examination revealed a fixed, hard, 3×2-cm mass with a smooth surface in the left parotid region. There was no palpable lymph node in the parotid gland or on the left side of the neck. Systemic physical and laboratory examinations revealed no abnormalities. Echography of the neck revealed an area of mixed echoes in the left parotid gland. Computed tomography (CT) demonstrated a 3×2-cm solid lesion in the left parotid gland and a 4.5×4.5-cm mass in the region of thymus. Radical resection of the parotid tumor and thoracotomy resection of the thymic tumor were performed.

### Tissue staining

The specimen was fixed in neutral buffered formalin and routinely processed with tissue sections embedded in paraffin. The sections were cut into 4-μm slices and stained with hematoxylin and eosin (H&E) for conventional evaluation. In addition to H&E, the following immunostains and special tissue stains were used: Cytokeratin (CK, AE1/AE3; Dako, Carpinteria, CA, USA), carcinoembryonic antigen (CEA; Zymed, San Francisco, CA, USA), p53 (Dako), S-100 (4c4.9; Zymed), Ki-67 (Dako) and phosphotungstic acid-hematoxylin (PTAH; Shanghai Lanji Science and Technology Co., Ltd., Shanghai, China).

### Macroscopic findings

**Figure 1:**
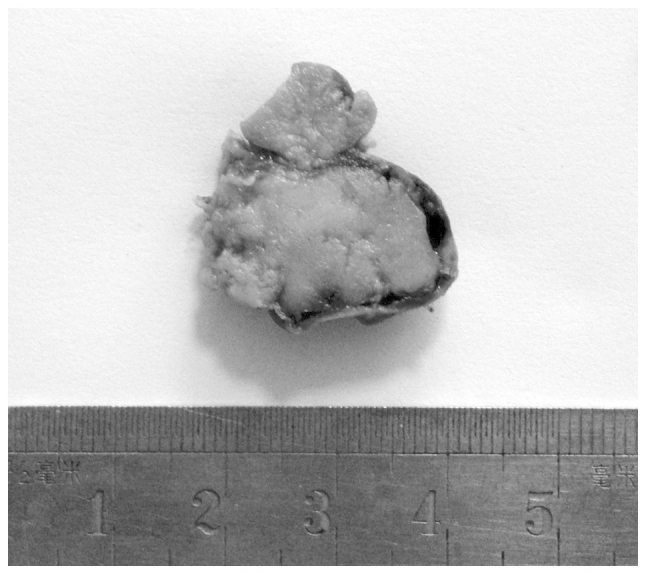
The cut surface of the parotid tumor, which was solid.

**Figure 2:**
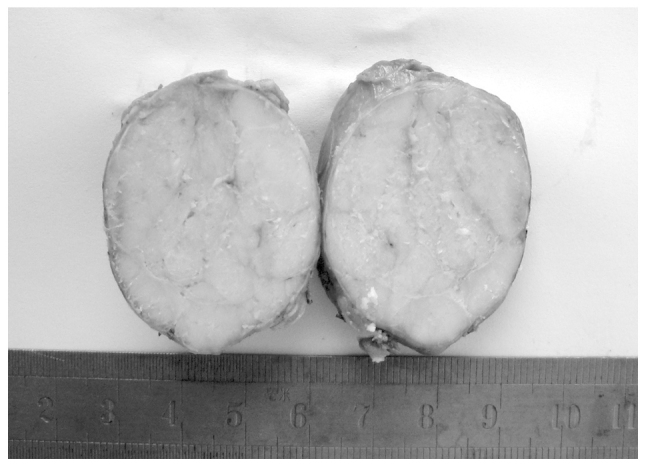
The tumor of the thymus was encapsulated and its cut surface was and solid.

The parotid tumor consisted of unencapsulated, irregular, cord-like, tan to gray masses. The cut surface was light brown, solid, and non-homogeneous with cystic degeneration, necrosis or hemorrhage (Fig. 1). The tumor of the thymus was encapsulated and its cut surface was solid and light brown (Fig. 2).

### Microscopic findings and immunohistochemistry

**Figure 3:**
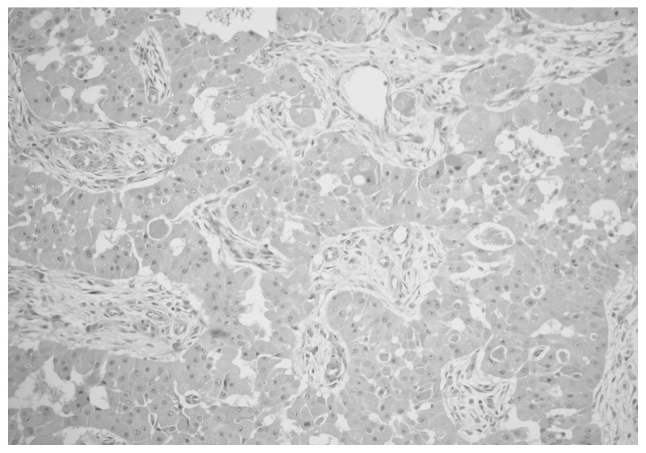
Oncocytic carcinoma of the parotid gland (stain, hematoxylin and eosin; magnification, ×200).

**Figure 4:**
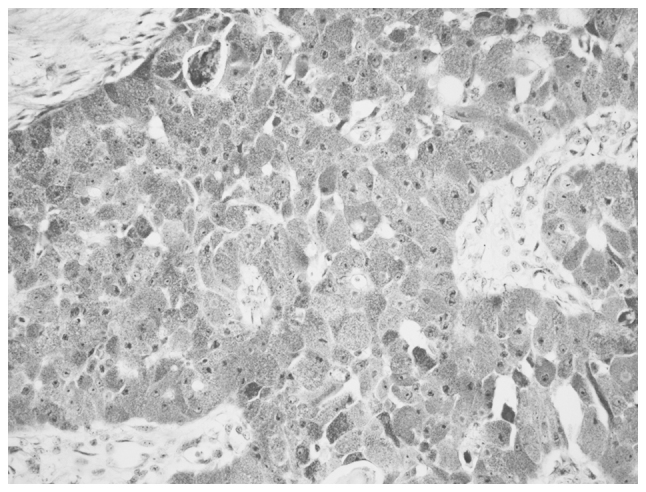
Mitochondria of the oncocytic carcinoma cells were stained (stain, phosphotungstic acid-hematoxylin; magnification, ×400).

**Figure 5:**
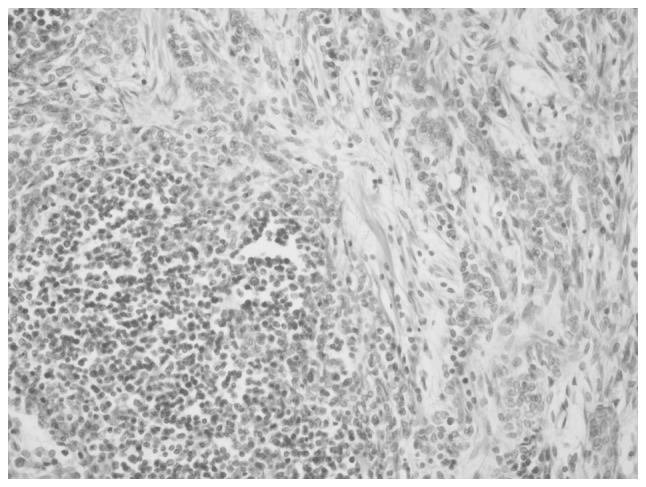
Thymoma, AB type, exhibiting type A with B-like features (stain, hematoxylin and eosin; magnification, ×200).

The parotid tumor had replaced a large area of the parotid gland, but perineural invasion and vascular invasion were not found. Neoplastic elements were large, round or polyhedral cells and were arranged in solid sheets, islands and cords. The cytoplasm was abundant, eosinophilic and finely granular. The nuclei were large and centrally or peripherally located, and the nucleoli were distinct and large (Fig. 3). PTAH staining distinctly illustrated positive, small, dark-blue cytoplasmic granules, which represented mitochondria (Fig. 4). Tumor cells were positive for CK, CEA, S-100 and p53 by immunohistochemistry. Additionally, PTAH staining illustrated positive dark-blue cytoplasmic granules. The tumor of the thymus consisted of a homogeneous population of neoplastic epithelial cells that were spindle- or oval-shaped and lacked nuclear atypia, admixed with foci rich in lymphocytes. The segregation of the two patterns was sharp and distinct (Fig. 5).

## Discussion

Oncocytes are large, granular, eosinophilic epithelial cells that are primarily found in glandular tissue, including that of the salivary glands and thyroid. In 1931, the pathologist Hamperl (26) used the term ‘oncocyte’ for this distinctive and typical cell type, which was taken from the Greek word ‘onkousthai’ (27). Normal oncocytes are observed in the salivary glands of aged patients and are considered to represent an age-related metaplasia or degenerative process (28). In salivary gland ductal epithelium, the appearance of oncocytes (oncocytic metaplasia) is rare prior to the age of 50; however, it is nearly universal beyond age 70. In 1989, Linnane *et al* (29) hypothesized that aging is the accumulation of mtDNA errors that lead to mitochondrial ‘respiratory failure’ and multisystem degeneration.

According to the World Health Organization histologic classification of salivary gland tumors (30), parotid oncocytic neoplasms are divided into three categories, including oncocytosis, oncocytoma and OC. OC has been given several names in the past, including oncocytic adenocarcinoma, malignant oncycytoma and malignant oxyphilic adenoma. Sugimoto *et al* (31) reported that OC commonly presents as a parotid mass with pain and facial nerve paralysis, and that such symptoms were observed in one of three patients with OC. However, the primary symptom in the patient reported in the current study was a slowly progressive, painless mass. Oncocytic carcinomas appear to arise from benign oncocytomas; however, they may arise *de novo* (30). In the current case, the malignant nature of the neoplasm was evidenced by the regional and distant lymph node metastases. No perineural invasion or infiltration of subcutaneous tissue was observed. Criteria for the diagnosis of oncocytic carcinoma of the salivary gland include: i) distant metastasis; ii) local lymph node metastasis; iii) perineural, vascular, or lymphatic invasion; and iv) frequent mitoses and cellular pleomorphism with extensive invasion and destruction of adjacent structures (32).

It has been reported that OC occurs predominately in the parotid gland of older adults with a mean age of occurrence of 62.5 years, and two-thirds of all cases occurring in males (30). We reviewed previous literature from the past 15 years (Table I) and found only 32 cases of parotid OC. In the current case, the patient age (historically ranging from 41 to 86 years with a median age of 62.5 years) and tumor location (historically 62.5% in the parotid gland) were in agreement with those of the previous reports.

Oncocytic carcinoma can be differentiated from benign oncocytoma, since the former includes distant metastases; local lymph node metastases; perineural, intravascular, or lymphatic invasion; and mitoses and cellular polymorphisms with destructive invasion of adjacent structures. Ki-67 immunostaining has been proposed as a tool for distinguishing OC from oncocytomas (33). In a previous study, the frequency of Ki-67 positive cells with nuclear staining was higher in OC compared to oncocytomas (34).

In contrast to oncocytic carcinoma, salivary duct carcinoma forms duct-like spaces with papillary and cribriform growth, and displays comedonecrosis (2). In addition, the presence of numerous mitochondria in the cytoplasm of the oncocytes, as confirmed by ultrastructural examination, is not found in the neoplastic cells of the other malignancies mentioned above, which can also be used for adjuvant diagnosis. However, the processes of fixing or embedding the specimens for light microscopy often destroys the fine structure of organelles in the cytoplasm, making it difficult to observe mitochondria clearly.

Acinic cell adenocarcinoma may be differentiated from oncocytic carcinoma by its amphophilic or basophilic cytoplasmic granules, negative staining for mithochondrial antigens and the presence of a connective tissue capsule. Cytologic examination of Warthin’s tumor shows oncocytes together with lymphocytes, amorphous material and cystic fluid. However, the possibility of oncocytoma should be considered when the smear contains only oncocytes (35).

PTAH staining has been successfully used to identify oncocytes; Brandwein and Huvos (36) particularly recommended the use of prolonged (48 h) PTAH staining, which results in positive, dark-blue cytoplasmic granules. It has also been reported that immunohistochemistry using an anti-mitochondrial antibody is a highly sensitive and specific method for identifying mitochondria using light microscopy (37).

Surgical excision is the most widely accepted method of treatment for OC (15), and the majority of the cases described in the literature have included neck dissection. Goode and Corio (38) reported that patients with tumors <2 cm in diameter appeared to have a better prognosis than those that were larger. Adjuvant radiotherapy has been used for the treatment of oncocytic carcinoma, but its true contribution has not yet been elucidated. OC has a potential risk of distant metastasis, and lung, liver and brain metastases have been reported (32). The long-term survival of patients with OC is poor due to distant disease, and long-term follow-up is necessary after therapy (2). In the current study, the patient not only had OC but also thymoma, which is exceedingly rare and may represent the first documented case in the literature.

## Figures and Tables

**Table I:** Reports of oncocytic carcinomas in the salivary gland.

Author (ref)	Age	Sex	Site	Size (cm)	Rec.	LM
Guclu (4)	65	F	P	3	Y	N
Mizutari (5)	55	M	Sm	3	N	N
Kimura (6)	61	M	P	4	N	Y
Wischerath (7)	59	M	Sm	2	N	Y
Lombardi (8)	45	M	Oth	N	N	N
Sugiyama (9)	84	M	Oth	4	N	N
Ardekian (10)	64	M	P	8	N	N
Cinar (11)	48	F	P	6	N	Y
Muramatsu (12)	82	M	Sm	4.5	N	Y
Ozawa (13)	58	M	P	3	N	Y
Nakada (14)	68	M	Sm	3	N	Y
Corbridge (15)	57	M	P	4	N	Y
Yang (16)	64	M	Sm	3.8	N	Y
Wang (17)	73	M	P	3	N	Y
Tian (18)	66	M	P	3	Y	N
Dong (19)	57	M	Sm	3	N	N/A
Zhou (20)	60	M	Oth	3.5	Y	Y
	57	M	P	7	N	Y
	48	M	P	3	N	N
	59	M	P	8	N	Y
	75	M	P	3	Y	Y
	68	M	P	4	Y	N
	41	M	P	3	N	N
	55	M	P	2.5	N	Y
	67	F	P	3.5	Y	N
	86	M	P	1	Y	N
	51	F	Oth	4	Y	Y
	68	M	P	3	Y	N
Lee (21)	51	M	Sm	3	N	Y
Gallego (22)	65	M	P	2.5	N	Y
Present case	66	M	P	2.5	N	Y

M, male; F, female; P, parotid; Sm, submaxillary salivary gland; Oth, other salivary gland; LM, lymph node metastasis; Rec., recurrence; N/A, not available; Y, yes; N, no.
